# Hematological analysis of naturally infecting blood parasites in dogs

**DOI:** 10.14202/vetworld.2023.681-686

**Published:** 2023-04-04

**Authors:** Worakan Boonhoh, Narin Sontigun, Punpichaya Fungwithaya, Tuempong Wongtawan

**Affiliations:** 1 Small Animal Research Group, Akkhraratchakumari Veterinary College, Walailak University, Nakhon Si Thammarat 80160, Thailand; 2 One Health Research Center, Walailak University, Nakhon Si Thammarat, 80160, Thailand; 3 Center of Excellence in Innovation on Essential Oil and Bioactive Compounds, Walailak University, Nakhon Si Thammarat, 80160, Thailand; 4 Center of Excellence Research for Melioidosis and Other Microorganisms, Walailak University, Nakhon Si Thammarat 80160, Thailand

**Keywords:** blood parasite, dogs, hematology, multiple blood parasite infection, tick-borne pathogens

## Abstract

**Background and Aim:**

Blood parasite infections such as anaplasmosis, babesiosis, and ehrlichiosis are commonly found in domestic dogs, which adversely influence their health. Many dogs are infected with multiple blood parasites that cause more severe diseases than a single infection. This study aimed to investigate the effect of multiple blood parasite infections on the hematological profiles of dogs at a shelter in Southern Thailand.

**Materials and Methods:**

The blood samples from 122 dogs were collected to assess the hematology profiles of uninfected, single-infected, and multiple blood parasite-infected dogs. The results were compared using Kruskal–Wallis test and Dwass–Steel–Critchlow–Fligner pairwise comparisons. The infections were confirmed by polymerase chain reaction.

**Results:**

The results showed that all the infected dogs had significantly lower red blood cell (RBC) count, hemoglobin (HB), hematocrit (HCT), and platelet count (PLT) than the uninfected dogs. Although the dogs with triple infection had lower RBC, HB, HCT, and PLT than those with double and single infections, the difference was not statistically significant.

**Conclusion:**

We proposed that triple blood parasite infection with *Anaplasma platys*, *Babesia vogeli*, and *Ehrlichia canis* caused more severe disease than double and single infections. Evaluating the hematological profiles of dogs naturally infected with single, double, and multiple blood parasite infections without clinical signs can enhance their health and welfare.

## Introduction

Ehrlichiosis, babesiosis, anaplasmosis, hepatozoonosis, and trypanosomiasis are serious vector-borne diseases caused by blood parasites in Thailand. Although these diseases are commonly found in animals [1–6], they can occasionally infect humans [7, 8]. Infections with *Babesia vogeli*, *Ehrlichia canis*, *Anaplasma platys*, *Hepatozoon canis*, and *Trypanosoma* spp., regularly observed in dogs in Thailand [4, 9, 10], share a vector, the brown dog tick (*Rhipicephalus sanguineus*), a common tick species [11, 12].

Transmission of tick-borne diseases is increasing across Thailand and Southeast Asia [4, 9–12] and is a significant threat to the health of animals, their companions, or even livestock [4, 9, 10, 13]. The clinical signs commonly observed in blood parasite diseases in dogs include fever, anorexia, weight loss, pale mucous membranes, and lymph node enlargement. Although *A. platys* rarely cause clinical disease, it can coinfect with other pathogens [14]. Microscopic examination, commercial test kits, and polymerase chain reaction (PCR) are generally used to detect blood parasites in dogs. Polymerase chain reaction-based methods are more sensitive and specific than other methods [4, 10], especially for *B. vogeli* and *E. canis* [10].

Single infections with blood parasites affect the health of dogs, causing anemia and, occasionally, thrombocytopenia and leukopenia [6, 9, 14–20]. The incidence of multiple blood parasite infections is increasing among dogs (up to 36%), particularly in tropical regions in developing countries [10, 11, 21–23]. However, studies investigating the hematological profiles of co-infection or multiple infections with tick-borne pathogens in dogs are limited [3, 24–26], especially in endemic areas, including Thailand. There are approximately 30 dog shelters in Thailand, with an average dog population of 500 in each shelter [10]. Due to the challenges in managing the health of the large dog population, these shelters have become “hubs” of blood parasite diseases. Most dogs in the shelters appear normal without clinical signs of blood parasite diseases unless they are severely infected [10].

Therefore, evaluating the hematological profile to detect multiple blood parasite infections in dogs might improve the diagnosis, prognosis, and treatment plan. This study aimed to compare the effects of single, double, and triple blood parasite infections on the hematological profile of naturally infected dogs.

## Materials and Methods

### Ethical approval

This study was approved by the Institutional Animal Care and Use Committee of Walailak University (WU-AICUC-63-036).

### Study period and location

The blood samples were collected in March 2021 at a dog shelter in Nakhon Si Thammarat province, Thailand. The samples were processed at Small Animal Teaching Hospital Laboratory, Akkhraratchakumari Veterinary College and Research Institute for Health Sciences laboratory, Walailak University.

### Animals and blood samples

The total dog population in the dog shelter was 487. Blood was collected from approximately 25% of the dogs (122 dogs). The inclusion criteria consisted of healthy dogs without any apparent external clinical signs. Their blood samples were collected from their cephalic veins and transferred into 3 mL ethylenediaminetetraacetic acid (EDTA) tubes for complete blood count and PCR analysis. Approximately 80% of the dogs in the shelter were female (389 from 487 dogs). Most dogs were mongrels and neutered. The shelter was an open-air style with 50% roof covering. The dogs were given adequate food and water. All the dogs were annually vaccinated with combination vaccines (distemper, adenovirus Type 2, parainfluenza, parvovirus, leptospira, coronavirus, and rabies) and injected ivermectin regularly to prevent ecto- and endo-parasites.

### Hematological analysis and blood pathogen detection

Hematological analysis was performed using an automated machine Procyte Dx (IDEXX Laboratories, Maine, USA). The parameters included red blood cell (RBC), hemoglobin (HB), hematocrit (HCT), mean corpuscular volume, mean corpuscular HB, mean corpuscular hemoglobin concentration (MCHC), RBC distribution width (RDW), white blood cell (WBC), neutrophil, lymphocyte, eosinophil, monocyte, and platelet counts (PLTs). Five blood parasite species, including *B. vogeli*, *E. canis*, *Hepatozoon canis*, *A. platys*, and *Trypanosoma* spp., were screened using 10% Giemsa-stained thin blood smear under 1000× magnification of a light microscope (Olympus, Tokyo, Japan) and confirmed using conventional PCR. The primers for the detection of blood pathogens were used as previously described [27–30].

For PCR analysis, DNA was extracted using the E.Z.N.A.^®^ Blood DNA Kit (Omega Bio-Tek, Norcross, GA, USA) according to the manufacturer’s instructions, and its concentration was measured using the Nano-Drop™ spectrophotometer (ThermoFisher Scientific, MA, USA). The PCR reaction mix contained 6.25 μL DreamTaq Green Master Mix (2×) (Thermo Scientific, Vilnius, Lithuania), 1–2 μL DNA template (100–200 ng/uL), 0.5 μL primer (0.4 μM) and nuclease-free water to a final volume of 12.5 μL, and was conducted using the Mastercycler Pro S machine (Eppendorf AG, Hamburg, Germany). The cycling conditions were as follows: Initial denaturation step at 95°C for 3 min, denaturation at 95°C for 30 s, and annealing at 54°C (for *B. vogeli, E. canis*, and *H. canis*) or 58°C (for *A. platys* and *Trypanosoma* spp.) for 30 s, extension at 72°C for 1 min for 35 cycles; and a final extension at 72°C for 5 min. The positive control included the DNA of known blood parasites from our previous study [10], while the negative control was nuclease-free water. The PCR products were visualized on a 1.5% agarose gel in 1× Tris acetate EDTA buffer and stained with SERVA DNA Stain G (SERVA, Heidelberg, Germany) under UV light with the ChemiDoc™ Imaging System (Bio-Rad, CA, USA).

### Statistical analysis

The data were analyzed using Jamovi 2.3.9 software (The Jamovi project, Sydney, Australia). The data were represented as mean ± standard deviation. The hematological parameters were compared and statistically analyzed using the Kruskal–Wallis test and Dwass–Steel–Critchlow–Fligner pairwise comparisons. p < 0.05 was considered statistically significant.

## Results

**Figure-1:**
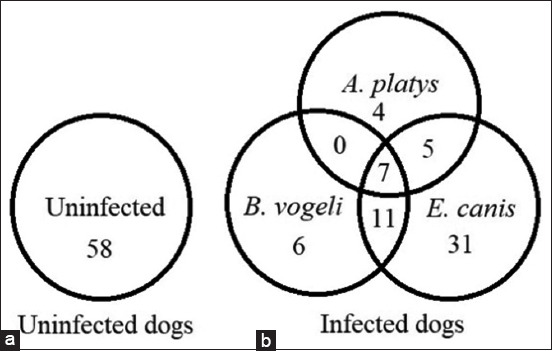
Venn diagram shows the number of dogs in this study. (a) 58 uninfected dogs and (b) 64 blood parasite infected dogs. *A. platys*: *Anaplasma platys*, *B. vogeli*: *Babesia vogeli*, *E. canis*: *Ehrlichia canis*.

Of the 122 dogs tested, 58 were uninfected, while the remaining (n = 64, 52.46%) were infected with at least one of these parasites; *A. platys*, *E. canis*, or *B. vogeli* (Figure-1). None of the dogs were infected with *H. canis* and *Trypanosoma* spp. The mean body score was 3/5, and the mean weight was approximately 10 kg. Most dogs in the study were female (n = 104, 85.25%).

### Single blood parasite infection

The average RBC, HB, and HCT of single-infected dogs were lower than that of the uninfected dogs. *Anaplasma platys*-infected dogs had the lowest HB and HCT, while the RBC, HB, and HCT values of *E. canis-*infected dogs were significantly lower than those of the uninfected dogs (Table-1).

**Table-1:** Hematological analysis of single blood parasite-infected dogs (*Anaplasma platys*, *Babesia vogeli*, and *Ehrlichia canis*) compared to uninfected dogs (Mean ± SD).

Parameters	Unit	Uninfected	*Anaplasma platys*	*Babesia vogeli*	*Ehrlichia canis*
RBC	(10^6^/μL)	6.40^a^ ± 0.97	5.26^a,b^ ± 0.72	6.17^a,b^ ± 1.32	5.64^b^ ± 1.57
HB	(g/dL)	13.90^a^ ± 2.44	11.40^a,b^ ± 1.76	13.30^a,b^ ± 3.07	12.10^b^ ± 3.21
HCT	(%)	43.00^a^ ± 7.39	35.00^a,b^ ± 4.83	42.50^a,b^ ± 7.87	37.30^b^ ± 8.86
MCV	(fL)	67.10 ± 5.52	67.30 ± 4.03	69.80 ± 9.11	67.30 ± 6.61
MCH	(pg)	21.80 ± 1.60	21.60 ± 0.49	21.70 ± 1.63	21.70 ± 1.55
MCHC	(d/dL)	32.50 ± 1.73	32.30 ± 2.23	31.20 ± 2.64	32.30 ± 1.97
RDW	(%)	14.20 ± 1.54	14.20 ± 1.56	15.20 ± 1.83	15.10 ± 2.34
WBC	10^3^ cells/μL	13.58 ± 4.96	15.40 ± 4.15	10.90 ± 2.55	11.85 ± 4.19
NEU	10^3^ cells/μL	8.65^a^ ± 3.84	7.82^a,b^ ± 0.57	6.21^a,b^ ± 1.78	6.03^b^ ± 2.32
LYM	10^3^ cells/μL	4.13 ± 2.74	6.77 ± 3.87	4.02 ± 2.56	4.80 ± 3.94
EOS	10^3^ cells/μL	0.25 ± 0.29	0.42 ± 0.38	0.18 ± 0.10	0.18 ± 0.15
MONO	10^3^ cells/μL	0.48 ± 3.15	0.24 ± 0.21	0.42 ± 0.13	0.77 ± 1.28
PLT	10^3^ cells/μL	127.74^a^ ± 64.35	83.25^a,b^ ± 48.54	89.33^a,b^ ± 84.68	70.83^b^ ± 39.79

^a,b^Different letters represent significant differences (p < 0.05), RBC=Red blood cell, HB=Hemoglobin, HCT=Hematocrit, MCV=Mean corpuscular volume, MCH=Mean corpuscular hemoglobin, MCHC=Mean corpuscular hemoglobin concentration, RDW=Red blood cell distribution width, WBC=White blood cell, NEU=Neutrophil, LYM=Lymphocyte, EOS=Eosinophil, MONO=Monocyte, PLT=Platelet count, SD=Standard deviation

The average PLT was lower in all single-infected groups, with the lowest count seen in *E. canis*-infected dogs (p < 0.001) than in the uninfected dogs. The neutrophil count of *E. canis*-infected dogs was significantly lower than that of uninfected dogs. The lymphocyte counts were higher in the *E. canis-* and *A. platys*-infected dogs than the uninfected ones but within the normal range. The highest lymphocyte count was seen in *A. platys-*infected dogs without any significant difference. The eosinophil count was higher in the *A. platys-*infected dogs than the uninfected dogs but within the normal range. The monocyte counts were highest in *E. canis-*infected dogs but within the normal value (Table-1).

The thrombocyte and PLTs of the dogs infected with these three blood parasites were approximately 30%–40% lower than uninfected dogs. In contrast, *A. platys* had more effect on HB than other blood parasites.

### Multiple blood parasite infections

All the infection groups had significantly lower RBC, HB, HCT, and PLTs than uninfected dogs. The lowest RBC, HB, HCT, MCHC, and PLTs were seen in the triple-infected dogs compared to the double and single infections. The severity of anemia (low RBC, HB, HCT) and thrombocytopenia decreased from multiple infections to single and non-infection, respectively (Table-2). We did not observe a double infection with *A. platys* and *B. vogeli*. Dogs with double infections with *A. platys* and *E. canis*, and *B. vogeli* and *E. canis* did not exhibit any significant difference compared to the uninfected dogs, except the RDW in *B. vogeli* and *E. canis* co-infection was significantly lower than the uninfected group (p < 0.001) (Table-3).

**Table-2:** Hematological analysis of uninfected, single, double, and triple-infected dogs (*Anaplasma platys*, *Babesia vogeli*, and *Ehrlichia canis*) shown as Mean ± SD.

Parameters	Unit	Uninfected	Single infection	Double infection	Triple infection
RBC	(10^6^/μL)	6.40^a^ ± 0.97	5.68^b^ ± 1.47	5.34^b^ ± 1.31	4.79^b^ ± 0.93
HB	(g/dL)	13.90^a^ ± 2.44	12.20^b^ ± 3.06	11.40^b^ ± 2.80	10.70^b^ ± 2.43
HCT	(%)	43.00^a^ ± 7.39	37.90^b^ ± 8.52	36.30^b^ ± 8.31	32.70^b^ ± 8.67
MCV	(fL)	67.10 ± 5.52	64.60 ± 6.72	69.20 ± 9.68	68.00 ± 5.07
MCH	(pg)	21.80 ± 1.60	21.70 ± 1.47	21.50 ± 1.33	22.30 ± 2.05
MCHC	(d/dL)	32.50^a^ ± 1.73	32.10^a^ ± 2.08	31.50^a,b,c^ ± 2.98	28.50^b,c^ ± 11.35
RDW	(%)	14.20^a^ ± 1.54	15.00^a^ ± 2.19	16.60^b^ ± 2.61	14.50^a,b^ ± 1.88
WBC	10^3^ cells/μL	13.58 ± 4.96	12.06 ± 4.08	13.39 ± 4.136	13.14 ± 3.05
NEU	10^3^ cells/μL	8.65^a^ ± 3.84	6.24^b^ ± 2.19	7.29^a,b^ ± 2.58	7.06^a,b^ ± 1.31
LYM	10^3^ cells/μL	4.13 ± 2.74	4.88 ± 3.75	5.19 ± 2.18	5.53 ± 2.18
EOS	10^3^ cells/μL	0.25 ± 0.29	0.21 ± 0.19	0.33 ± 0.42	0.24 ± 0.12
MONO	10^3^ cells/μL	0.48 ± 3.15	0.67 ± 1.13	0.52 ± 0.33	0.28 ± 0.20
PLT	10^3^ cells/μL	127.74^a^ ± 64.35	74.75^b^ ± 48.08	78.00^b^ ± 70.44	59.71^b^ ± 32.77

^a,b,c^Different letters represent significant differences (p < 0.05), RBC=Red blood cell, HB=Hemoglobin, HCT=Hematocrit, MCV=Mean corpuscular volume, MCH=Mean corpuscular hemoglobin, MCHC=Mean corpuscular hemoglobin concentration, RDW=Red blood cell distribution width, WBC=White blood cell, NEU=Neutrophil, LYM=Lymphocyte, EOS=Eosinophil, MONO=Monocyte, PLT=Platelet count, SD=Standard deviation

**Table-3:** Hematological analysis of uninfected and coinfected dogs (*Anaplasma platys*, *Ehrlichia canis* co-infection, *Babesia vogeli*, and *Ehrlichia canis* co-infection) shown as Mean ± SD.

Parameters	Unit	Uninfected	*Anaplasma platys* and *Ehrlichia canis*	*Babesia vogeli* and *Ehrlichia canis*
RBC	(10^6^/μL)	6.40 ± 0.97	5.35 ± 1.34	5.33 ± 1.36
HB	(g/dL)	13.90 ± 2.44	11.20 ± 2.87	11.50 ± 2.91
HCT	(%)	43.00 ± 7.39	33.80 ± 7.90	37.40 ± 8.61
MCV	(fL)	67.10 ± 5.52	63.90 ± 4.80	71.60 ± 10.54
MCH	(pg)	21.80 ± 1.60	21.00 ± 1.07	21.80 ± 1.40
MCHC	(d/dL)	32.50 ± 1.73	33.00 ± 1.00	30.80 ± 3.36
RDW	(%)	14.20^a^ ± 1.54	15.20^a,b^ ± 1.07	17.30^b^ ± 2.87
WBC	10^3^ cells/μL	13.58 ± 4.96	13.67 ± 3.24	13.27 ± 4.63
NEU	10^3^ cells/μL	8.65 ± 3.84	7.59 ± 1.99	7.16 ± 2.88
LYM	10^3^ cells/μL	4.13 ± 2.74	5.15 ± 3.09	5.20 ± 2.45
EOS	10^3^ cells/μL	0.25 ± 0.29	0.29 ± 0.19	0.35 ± 0.48
MONO	10^3^ cells/μL	0.48 ± 3.15	0.58 ± 0.47	0.49 ± 0.27
PLT	10^3^ cells/μL	127.74 ± 64.35	78.40 ± 56.99	77.82 ± 78.38

^a,b^Different letters represent significant differences (p < 0.05), RBC=Red blood cell, HB=Hemoglobin, HCT=Hematocrit, MCV=Mean corpuscular volume, MCH=Mean corpuscular hemoglobin, MCHC=Mean corpuscular hemoglobin concentration, RDW=Red blood cell distribution width, WBC=White blood cell, NEU=Neutrophil, LYM=Lymphocyte, EOS=Eosinophil, MONO=Monocyte, PLT=Platelet count, SD=Standard deviation

There was no significant difference in the WBC count in all the groups. Neutrophil count in single infection was the lowest among all infection groups, which was significantly lower than in uninfected dogs. Higher lymphocyte counts were observed in single, double, or triple infections without any significant difference. Monocyte counts in triple infection and eosinophil counts in single infection were lower than in the other groups, without any significant difference.

## Discussion

### Single blood parasite infection

We observed that canine anaplasmosis is associated with anemia in Thailand [16, 18], unlike previously reported [17]. We also found that it is associated with thrombocytopenia, which differs from other studies [16, 18]. This pathogen is unique because it is the only intracellular pathogen described in humans or animals that specifically infects platelets. In other countries, anemia and thrombocytopenia were also observed [13–15, 19].

Consistent with the previous studies, we showed that *E. canis* infection mainly causes leukopenia in Bangkok and Kanjanburi [20, 24, 31] but also causes higher neutrophil counts than healthy dogs in Southern Thailand [9]. We also showed extremely low PLTs in *E. canis*-infected dogs, which were over 50% lower than that reported previously from a neighboring area [9] but were within normal values [32].

Lymphopenia was observed in dogs infected with *B. vogeli* in Songkhla, Bangkok, and Kanchanaburi [9, 20], but not in our study. These might be associated with limited *B. vogeli* infections through randomized sampling. None of the dogs showed clear signs of babesiosis.

### Multiple blood parasite infection

Two reports from Thailand with different types of co-infection data showed that multiple infections significantly reduce the RBC, HB, HCT, and PLTs [20, 24]. Another study showed that co-infection with *B. vogeli* and *E. canis* exacerbates the disease, which showed lower RBC, HB, HCT, and lymphocyte counts than single *B. vogeli* infection [26]. A previous study showed that dogs coinfected with *A. platys* and *E. canis* displayed lower HCT and PLTs than single-infected ones [25]. As blood parasite infections might have a dose-dependent correlation with anemia, triple blood parasite infections reduce the RBC, HB, HCT, and platelet values. The PLT in uninfected dogs was slightly lower than in other areas of Thailand [9, 20, 24, 31]. Moreover, the dogs infected with either single or multiple blood parasites showed moderate to severe thrombocytopenia with no apparent external clinical signs.

Blood parasite-infected dogs showed lower lymphocyte count. Canine monocytic ehrlichiosis (CME) showed a higher correlation with T- and cytotoxic T (Tc) cells, and the absolute number of Tc cells was higher in peripheral blood. The percentage of T helper cells and the absolute and relative values of B lymphocytes were higher in healthy dogs than in CME dogs [33]. Dogs naturally infected with *Babesia rossi* displayed significantly lower mean lymphocyte count and percentages of CD3+, CD4+, and CD8+ T lymphocytes than uninfected dogs [6]. Here, we also observed decreased lymphocyte counts in *B. vogeli-*infected dogs.

In this study, the infected dogs did not show obvious clinical signs of blood parasite infection by physical examination. However, their hematological analysis indicated significant anemia and thrombocytopenia. Treatment of multiple blood pathogen infections in dogs is more complicated and takes longer than a single infection. Our preliminary study data of multiple blood parasite infection treatment in dogs appear to be improved with the hematology profile and devoid of parasites in blood circulation when given 10 mg/kg doxycycline once a day for at least 8 weeks (Boonhoh *et al.*, unpublished data).

### Limitations of the study

Our study had some limitations. Due to the limited number of dogs, randomized group sampling, time of sampling, and limited sample collection location, we could not explore the hematological data of *A. platys* and *B. vogeli* co-infection and multiple infections with *H. canis* and *Trypanosoma* spp. Few *A. platys* and *B. vogeli*, single infection cases were detected in this study, which might not adequately represent the hematological profile of single blood parasite infection with *A. platys* and *B. vogeli* in dogs.

## Conclusion

There are limited studies on multiple blood pathogen infections in dogs. Based on the hematological profile of the tested dogs, we proposed that triple multiple blood parasite infections with *A. platys, B. vogeli*, and *E. canis* cause more severe disease than double and single infections. Further treatment guidelines for multiple blood parasite infections in dogs should be investigated to enhance the health and welfare of these animals.

## Authors’ Contributions

TW: Designed and supervised the study, participated in sample collection, and revised the manuscript. WB: Performed physical examinations, collected blood samples, analyzed data, and prepared and revised the manuscript. NS: Performed PCR and participated in sample collection, data analysis, and revised manuscript. PF: Participated in sample collection and data analysis. All authors have read, reviewed, and approved the final manuscript.
